# Vaccination against 2009 pandemic H1N1 in a population dynamical model of Vancouver, Canada: timing is everything

**DOI:** 10.1186/1471-2458-11-932

**Published:** 2011-12-14

**Authors:** Jessica M Conway, Ashleigh R Tuite, David N Fisman, Nathaniel Hupert, Rafael Meza, Bahman Davoudi, Krista English, P van den Driessche, Fred Brauer, Junling Ma, Lauren Ancel Meyers, Marek Smieja, Amy Greer, Danuta M Skowronski, David L Buckeridge, Jeffrey C Kwong, Jianhong Wu, Seyed M Moghadas, Daniel Coombs, Robert C Brunham, Babak Pourbohloul

**Affiliations:** 1 Division of Mathematical Modeling, University of British Columbia Centre for Disease Control, 655 West 12th Avenue, V5Z 4R4 Vancouver, British Columbia, Canada; 2 Department of Mathematics, University of British Columbia, Vancouver, British Columbia, Canada; 3 Dalla Lana School of Public Health, University of Toronto, Toronto, Ontario, Canada; 4 Departments of Public Health and Medicine, Weill Medical College of Cornell University, New York, NY, USA; 5 New York-Presbyterian Hospital, New York, NY, USA; 6 Department of Mathematics and Statistics, University of Victoria, Victoria, British Columbia, Canada; 7 Section of Integrative Biology, The University of Texas at Austin, Austin, TX, USA; 8 Department of Pathology and Molecular Medicine, McMaster University, Hamilton, Ontario, Canada; 9 Centre for Communicable Diseases and Infection Control, Public Health Agency of Canada, Toronto, Ontario, Canada; 10 Epidemiology Services, British Columbia Centre for Disease Control, Vancouver, British Columbia, Canada; 11 Surveillance Lab, Department of Epidemiology and Biostatistics, McGill University, Montreal, Québec, Canada; 12 Bureau de surveillance épidémiologique, Direction de santé publique de Montréal, Montréal, Québec, Canada; 13 Institute for Clinical Evaluative Sciences, Toronto, Ontario, Canada; 14 Centre for Disease Modelling, York University, Toronto, Ontario, Canada; 15 School of Population and Public Health, Faculty of Medicine, University of British Columbia, Vancouver, Canada

## Abstract

**Background:**

Much remains unknown about the effect of timing and prioritization of vaccination against pandemic (pH1N1) 2009 virus on health outcomes. We adapted a city-level contact network model to study different campaigns on influenza morbidity and mortality.

**Methods:**

We modeled different distribution strategies initiated between July and November 2009 using a compartmental epidemic model that includes age structure and transmission network dynamics. The model represents the Greater Vancouver Regional District, a major North American city and surrounding suburbs with a population of 2 million, and is parameterized using data from the British Columbia Ministry of Health, published studies, and expert opinion. Outcomes are expressed as the number of infections and deaths averted due to vaccination.

**Results:**

The model output was consistent with provincial surveillance data. Assuming a basic reproduction number = 1.4, an 8-week vaccination campaign initiated 2 weeks before the epidemic onset reduced morbidity and mortality by 79-91% and 80-87%, respectively, compared to no vaccination. Prioritizing children and parents for vaccination may have reduced transmission compared to actual practice, but the mortality benefit of this strategy appears highly sensitive to campaign timing. Modeling the actual late October start date resulted in modest reductions in morbidity and mortality (13-25% and 16-20%, respectively) with little variation by prioritization scheme.

**Conclusion:**

Delays in vaccine production due to technological or logistical barriers may reduce potential benefits of vaccination for pandemic influenza, and these temporal effects can outweigh any additional theoretical benefits from population targeting. Careful modeling may provide decision makers with estimates of these effects before the epidemic peak to guide production goals and inform policy. Integration of real-time surveillance data with mathematical models holds the promise of enabling public health planners to optimize the community benefits from proposed interventions before the pandemic peak.

## Background

The emergence of a novel swine-origin influenza A/H1N1 virus in the spring of 2009 led the WHO to declare the first influenza pandemic of the 21st century [1]. In the Canadian province of British Columbia, Canada it first appeared as a spring-summer wave of very low intensity, but resurged as a more substantial and widespread second wave in the fall, as in the rest of Canada and many other countries worldwide [2,3]. The commencement of this second wave varied by jurisdiction, likely depending in part upon prior first wave experience, demographic and environmental factors. In British Columbia, second wave pandemic H1N1 (pH1N1) activity began slowly in early September 2009, coinciding with the reconvening of schools and universities; increased more abruptly in mid-October; peaked during the last week of October; and fully resolved by the end of the calendar year [4]. This stands in marked contrast with Canada's usual influenza season, which typically spans November to April [5].

Characterization of the epidemiology of pH1N1 began early in the outbreak. One of the striking features of this novel influenza strain is its association with higher attack rates in younger individuals, compared to what is usually observed for seasonal influenza [6-12]. Proposed explanations for an apparently reduced susceptibility in older adults include pre-existing immunity due to prior exposure to related H1N1 strains circulating prior to 1957 [13], accumulated cross-protection derived from seasonal human H1N1 infection across the lifespan [14], and/or differences in the contact networks of younger individuals, who are more highly connected (and hence more likely to be exposed to pH1N1 early in an epidemic) than older individuals [15]. Regardless of the reasons underlying differential vulnerability to infection by age, this observation has important implications for the design and implementation of mitigation strategies for pH1N1 and future pandemic influenza viruses.

Vaccination is an important influenza control measure and was a key component of many countries' pandemic preparedness plans. Production of pH1N1 vaccine began soon after the pandemic potential of pH1N1 was recognized. However, the early arrival of the second wave of pH1N1 in many regions of the northern hemisphere, combined with production delays, resulted in the implementation of vaccination programs in populations already experiencing moderate to high incidence of pH1N1, a sequence of events expected to reduce the ultimate population impact of immunization. Quantifying this reduction and determining how it might have been mitigated through alternative dispensing schemes motivated the modeling effort reported here.

Seasonal influenza vaccination campaigns have historically targeted those at greatest risk of the severe outcomes of influenza--notably the very young, the elderly and individuals of all ages with underlying medical conditions, as well as their close contacts, including health care workers [5]. It has been suggested that vaccination of schoolchildren might be a more effective strategy [16], since younger age groups are responsible for a disproportionate amount of influenza transmission, and targeting these groups would indirectly protect at-risk groups [17,18]. Some regions--notably the province of Ontario--have adopted a universal influenza immunization program (UIIP) whereby influenza vaccine is provided free to all citizens over the age of 6 months [19]. In the case of pH1N1, the misalignment between vaccine availability and the onset and peak of the second pandemic wave in the fall 2009 required prioritization of vaccine. Greater infection risk and poor outcomes in younger individuals argued for targeted vaccination of younger age groups [18]. Conversely, older individuals were at decreased risk of infection with pH1N1, but experienced higher rates of severe outcomes, including mortality [20-22]. This is illustrated in outcome surveillance data from British Columbia for both waves, showing that pH1N1 detection was higher in children, but both the per-laboratory confirmed case hospitalization and fatality rates were greatest in older adults, with substantial increase beginning at age 50. The pH1N1 vaccine prioritization schemes adopted by many countries required a balance of these competing considerations and ultimately differed from seasonal influenza recommendations as a result of vaccine delay and unique pandemic patterns of age-related risk [12,23-26].

Mathematical models of the spread of the pH1N1 virus across the population played a prominent role in the assessment of the pH1N1 pandemic risk and in the evaluation and design of intervention and control strategies. During the early stages of the pH1N1 pandemic, mathematical analyses of the initial data from Mexico and other countries allowed researchers to estimate the transmissibility of the pH1N1 virus, as measured by the basic reproduction number [8,11,27,28]. As the pandemic progressed, many modeling studies investigated the impact of different kinds of containment strategies like social distancing [29], vaccination [18,28-30], and the use of antivirals [29,31]. These studies among many others provided important information to policy makers and demonstrated the value of mathematical modeling as a risk assessment tool during the emergence of new infectious disease agents.

We developed a mathematical model of the transmission dynamics of the pH1N1 virus in the Greater Vancouver Regional District (GVRD) to quantify the impact of vaccination campaign timing in relation to the pandemic peak on the projected outcomes of these strategies. Models incorporating age structure and/or heterogeneity in disease vulnerability have long been used to investigate issues surrounding immunization, either with instantaneous vaccinations (e.g. [18,32-34]) or with vaccinations concurrent with the epidemic (e.g. [35]). In particular such models have been used to investigate vaccination strategies in combination with campaign timing, as in [36-38]. Our aim was to develop a population dynamical model that represents the transmission of pH1N1 influenza in a realistic urban setting. We therefore incorporated into this model detailed demographic and behavioural factors that provide the basis for pandemic transmission dynamics. In particular, in addition to modeling the age structure of the population, we also considered the heterogeneity in the contact rates between individuals by age to better represent the overall contact structure of the population and better approximate the time course of the epidemic. To parameterize our model we used data from the Greater Vancouver Regional District (GVRD), British Columbia (BC), Canada. This model was used to assist policymakers in evaluating different intervention strategies throughout the Fall (2009) including the impact of vaccination of schoolchildren in addition to the specified target groups, social distancing, as well as assessing the likelihood of observing a third wave in the winter of 2010. However, as we discuss below, one should observe similar outcomes in most urban settings.

## Methods

### Model overview

We developed a susceptible-infected-recovered (SIR)-type compartmental model, extended to capture heterogeneity in age and behaviour, both of which affect contact patterns between individuals. A complete description of the model is provided in the Appendix, Additional file 1. The population was divided into six compartments representing different disease states: susceptible (S), vaccinated against pH1N1 (SW), exposed (E), pre-symptomatically infectious (before clinical infection onset) (A), infectious (either symptomatic, after clinical infection onset; or asymptomatic) (I), and immune (M). In an attempt to capture the social network-type dynamics in a more computationally tractable manner, the population was stratified by age and activity level. Activity level groupings correspond to the average number of contacts that individuals have per week. Mixing of the different age/activity groups was obtained from models of the GVRD contact network [39,40]. Each compartment is comprised of many sub-compartments for each age and activity level grouping. We ran the model to investigate the time period from September 1, 2009 until May 31, 2010.

## Model parameterization

Epidemiological parameters for pH1N1 were derived from the published literature and empirical data, with some assumptions reflecting the nature of influenza infection (Table 1). For simulations, the baseline transmission parameter values were: basic reproduction number *R*_0 _of 1.4, latent period of 3 days, and infectious period of 7 days (1 day before clinical infection onset and 6 days either symptomatic, after clinical infection onset, or asymptomatic) [21,41]. Demographic and behavioural data used to derive age-specific average number of contacts per week was obtained for the GVRD [39,42].

**Table 1:** Model parameter values

Variable	Age group	Value (range)	Source
Population size	0-2	63,025	2006 Census [42]
		
	3-4	42,260	
		
	5-17	322,670	
		
	18-24	203,500	
		
	25-54	975,875	
		
	55-64	237,795	
		
	≥65	271,455	
		
	Total	2,116,580	

Latent period (days)	All	3 (2-4)	Tuite et al. 2010 [22]

Initial asymptomatic infectious period (in all infected individuals) (days)	All	1	Liao et al. 2010 [43]

Total duration of infectiousness (days)	All	7 (5-7)	Tuite et al. 2010 [22]; De Serres [44]

Basic Reproduction number (*R*_0_)	All	1.4 (1.2-1.8)	Pourbohloul et al. 2009 [27]

Proportion of population with pre-existing immunity	≥55	0.5	Centers for Disease Control and Prevention 2009 [13]; Fisman et al. 2009 [6]

Vaccine efficacy	All	0.9 (0.5-1)	Product Monograph Arepanrix(tm) H1N1 [45]; Waddington et al., 2010 [46]

Proportion of infected individuals who self-isolate	All	0.1 (0.1-0.6)	Assumption

Mortality (per 100,000 infections)	0-2	30	Donaldson et al. 2009 [47]
		
	2-4	27	
		
	5-17	11	
		
	18-24	12	
		
	25-54	30	
		
	55-64	65	
		
	≥65	980	

### Initial condition

We set the start time of the epidemic to September 6th, which corresponds to the start of school in Vancouver. As of August 31st there had been only a total of 812 laboratory-confirmed cases of pH1N1 influenza since April of 2009 in British Columbia [48]. We assumed that the number of actual (currently infected) cases on September 6th was 100. We then distributed them through the age and behaviour compartments randomly with probably weighted by population fraction and contact rate in each compartment. Each result we show in the following represents the mean of 10000 simulations starting with different random initial conditions. We further assume that the rest of the population is completely susceptible. Although there was pH1N1 activity in the GVRD in Spring 2009 which would result in some background immunity, it was quite low, as evidenced by numbers of laboratory-confirmed cases and reported hospitalizations [48]. We therefore assumed the effect of background immunity was negligible.

### Vaccination implementation

The time to administer vaccine across the population was assumed to be 8 weeks. Vaccine distribution spanned this roll-out period and resulted in final coverage levels in different age groups (described below). For results shown below, we assumed the daily number of vaccinations gradually decreased throughout the campaign. However using different vaccination rates gave quantitatively and qualitatively similar results; see Appendix, Additional file 1 for details and additional information. We assumed that there was no intra-group age prioritization for vaccine distribution among those who were eligible to receive the vaccine. We assumed a 2-week delay between vaccine receipt and development of a protective immune response [49].

All individuals receiving vaccine were assumed to have a reduction in pH1N1 acquisition risk equal to 90% (modeled as a "leaky vaccine"). Although this number may seem high at first, studies of both the efficacy and the effectiveness of the pH1N1 vaccine used in Canada have shown remarkably high levels of protection [45,46,50]. In particular, initial studies of seroconversion and seroprotection rates by the adjuvanted pH1N1 vaccine used in Canada showed high levels (>90%) of both consistently across all age groups [45,46]. Further, a recent study with over 500 participants in Canada showed that this vaccine was highly effective at preventing laboratory-confirmed pH1N1 influenza [50]. The reported high vaccine protection (effectiveness), generally over 90%, was maintained across most sensitivity analyses [50]. These results are supported by a similar study investigating the effectiveness of a the same vaccine in children <10 years of age, which reported statistically significant 100% vaccine effectiveness for a single dose assuming a 2-week development of protective immune response [51]. We therefore assumed a baseline vaccine efficacy/effectiveness of 0.9 across all age groups. Although as mentioned here there is strong evidence that the protection offered by the pH1N1 vaccine predominantly distributed in Canada was extremely high, we nevertheless also performed extensive sensitivity analyses of our results and conclusions assuming much lower values of vaccine protection (see Figures S5 and S6, Additional file 1).

### Vaccination scenarios

We considered four different vaccination strategies. In the first two and fourth scenarios, the final population vaccination coverage was approximately 47%, matching age-standardized estimates of overall pH1N1 vaccine coverage in the GVRD. However, each of these scenarios simulated different patterns of vaccine distribution across age groups. The Actual Coverage (AC) strategy assumed a vaccine uptake in the different age groups corresponding to the observed uptake of pH1N1 vaccine in the GVRD during the Fall vaccination campaign of 2009, which covered an aggregate of 47% of the population (BC Centre for Disease Control, personal communication; see Table 2 for age-specific vaccination coverage). For the Uniform Coverage (UC) strategy, final vaccine uptake was set at 47% within each age group.

**Table 2:** Age-specific coverage levels for the different vaccination scenarios

Vaccination strategy	Age group	Vaccination coverage (%)
Actual pH1N1 vaccination in the Greater Vancouver Regional District (AC)	0-2	60.0
	
	3-4	60.0
	
	5-17	49.0
	
	18-24	36.1
	
	25-54	41.4
	
	55-64	47.4
	
	≥65, community-dwelling	58.8
	
	≥65, long-term care	58.8

Uniform coverage (UC)	All ages	47

Parents and children (PC)	5-17	100
	
	30-39	100
	
	All other ages	0

Parents and children/actual sequence (PC+)	0-2	35.0
	
	3-4	35.0
	
	5-17	100.0
	
	18-24	24.9
	
	25-54	33.8
	
	55-64	27.6
	
	≥65, community-dwelling	34.3
	
	≥65, long-term care	34.3

Because of heightened interest in prioritization of demographic groups potentially capable of accelerating early transmission of influenza [18], we also modeled a Parents and Children (PC) strategy in which 100% of children aged 5-17 and their parents (represented by 100% of adults aged 30-39) received the vaccine. No other members of the population were vaccinated under this strategy, which had a final population coverage of only 36% in contrast to the 47% of the first two scenarios, a difference that corresponds to over 230,000 fewer doses distributed throughout the GVRD.

Finally since the PC strategy has a lower population coverage than the first two, we formulated a fourth strategy that combines it with a more general vaccine distribution to attain a final 47% coverage. In the AC and UC coverage scenarios described above, 36% population coverage is achieved in approximately 38 days. For this fourth scenario, the parents-and-children/actual sequence strategy (PC+), we assume that after that point (38 days) the vaccine is then made available to the general public for the remaining time of the campaign. We model this using AC coverages scaled down proportionally to make up for the 11% deficit in coverage of the PC strategy.

To reflect actual pH1N1 response activities in GVRD, we initiated each of the modeled vaccination programs on October 26, 2009. For the baseline 8-week campaign length, vaccination was completed by the end of the week of December 14, 2009.

### Sensitivity analyses

We tested the robustness of projections to model assumptions by performing sensitivity analyses over plausible ranges of parameter values. A range of values for *R*_0_, latent period, infectious period, vaccine efficacy, and vaccination campaign lengths (see Table 1) were tested in the absence of vaccination (where appropriate) and in the presence of each of the three vaccination strategies. For each vaccination strategy, we also tested the effect of varying the start date of vaccination campaigns under baseline transmission parameter values. Model outputs were assessed for vaccination campaigns initiated at the beginning of each week from July 5, 2009, to November 22, 2009. Finally, we also assessed the impact of using different pH1N1 age-specific mortality profiles on our results.

## Results

### Baseline case without and with vaccination

**Figure 1:**
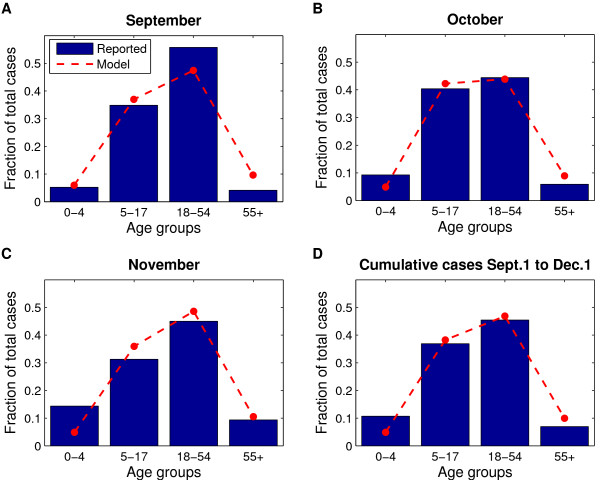
**Age distribution of reported cases and comparison to model predictions**. Population denominators for the given age groups were derived from 2006 census data for the GVRD [41]. **a **New infections in September 2009 only, **b **new infections in October 2009 only, **c **new infections in November 2009 only, and **d **cumulative number of infections, September 1-December 1, 2009. Vaccination began the week of October 26, 2009 and continued for 8 weeks, to obtain the actual coverage levels outlined in Table 2. The resulting epidemic curves assume *R*_0 _of 1.4, latent period of 3 days, and infectious period of 7 days.

**Figure 2:**
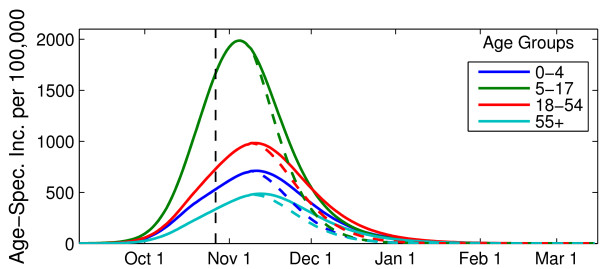
**Age-specific daily incidence of pH1N1 cases**. The number of new cases per day per 100,000 individuals is presented in the absence (*solid lines*) and presence (*dotted lines*) of pH1N1 vaccination. Vaccination began the week of October 26, 2009 and continued for 8 weeks, to obtain the actual coverage levels outlined in Table 2. The resulting epidemic curves assume *R*_0 _of 1.4, latent period of 3 days, and infectious period of 7 days.

Although true pH1N1 infection incidence is difficult to determine, the recorded spread of pH1N1 through different age groups in the GVRD starting in the early autumn of 2009 was closely reproduced by the model using the baseline parameter values for pH1N1 (Figure 1). In particular, the model predicted that the highest number of infections in the 18-54 age group, followed by the 5-17, 0-4, and ≥55 age groups (Figure 1d), which is similar to what was observed within laboratory-confirmed reported cases. Further, the model predicted a peak of pH1N1 activity in Vancouver in early November and highest *age-specific *attack rates in the 5-17 age group, followed by the 18-54, 0-4, and ≥55 age groups (Figure 2, dashed lines).

Figure 2 shows the impact of simulating the actual GVRD pH1N1 vaccination campaign to the baseline model (Actual Coverage strategy initiated October 26, 2009, dashed lines). This intervention reduced the simulated cumulative attack rate from 48.3% to 42.0%, representing over 120,000 pH1N1 infections prevented in the Vancouver population. The number of cases prevented is greatest in the 5-17 year old age group (7625 per 100,000 population), followed by the 18-54 (6011 per 100,000), 0-4 (5824 per 100,000), and ≥55 (4152 per 100,000) age groups. The fraction of cases prevented is not equivalent across age groups with this strategy: individuals aged 0-4 and ≥55 years experience the largest relative reduction in final attack rate (18% decrease), while those in the 5-17 age group have the smallest (10% decrease).

### Impact of timing of vaccination campaign on final attack rates

**Figure 3:**
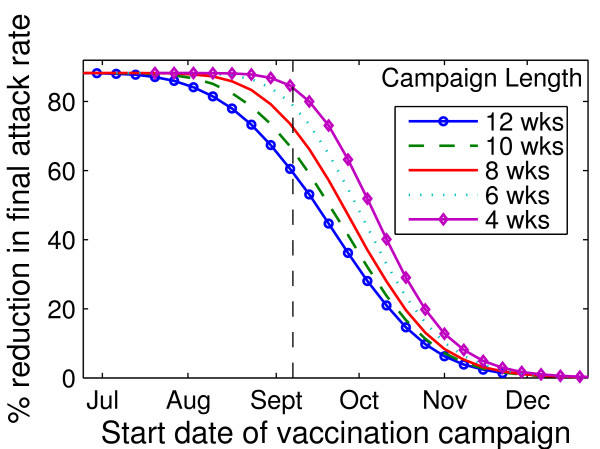
**Effect of vaccination campaign start date on overall attack rate**. For a given vaccination campaign start date, the percent reduction in final attack rate relative to that observed in the absence of vaccination is presented for campaign lengths of between 4 and 12 weeks. Vaccination campaigns were implemented weekly, starting July 5, 2009, with the last campaign started November 22, 2009. The start of the Vancouver influenza season on September 6, 2009 is indicated by a vertical line. All simulations assumed *R*_0 _of 1.4, latent period of 3 days, and infectious period of 7 days.

As expected, earlier implementation of the Actual Coverage strategy resulted in smaller final attack rates (Figure 3). Initiation of vaccination campaigns in the presence of moderate levels of circulating pH1N1, but prior to the epidemic peak, had a modest but detectable impact on final attack rates. For example, under baseline assumptions, an 8-week campaign initiated 2 weeks before epidemic onset (August 24) reduced the attack rate by approximately 83%; an 8-week campaign initiated 1 month into the epidemic (October 5) reduced the attack rate by approximately 47%. Additionally, distribution of vaccine in a shorter period of time resulted in a greater reduction in attack rates for a given vaccination campaign start date.

### Transmission and mortality impact of different vaccination strategies

**Figure 4:**
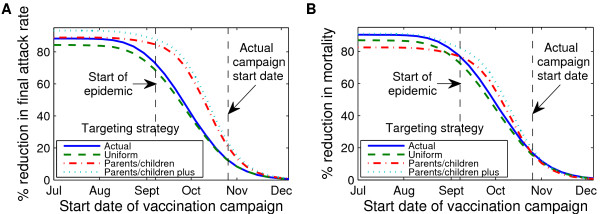
**Impact of timing on the effectiveness of different vaccination strategies**. Vaccination campaigns were implemented weekly, starting July 5, 2009, with the last campaign started November 22, 2009. For a given campaign start date, the reduction in **a **final attack rates and **b **mortality relative to no vaccination was assessed using actual (*blue, solid line*), uniform (*green, dashed line*), parents and children only (*red, dash-dotted line*), or parents and children only/actual sequence (*cyan, dotted line*) vaccination strategies. All simulations assumed *R*_0 _of 1.4, latent period of 3 days, infectious period of 7 days and an 8-week vaccine roll-out period.

Under baseline assumptions (i.e., vaccination initiated on October 26, 2009 with an 8-week campaign), vaccination of not only parents and children/general population in sequence (PC+), but also of parents and children (PC) alone were more effective than the actual coverage (AC) or a uniform coverage (UC) strategy in reducing the influenza attack rate but each achieved equivalent mortality reduction (see Tables 3 and 4, Figure 4a and 4b, and Figures S1 and S2, Additional file 1 for additional values of *R*_0_). Comparing the AC, UC PC, and PC+ strategies for different campaign initiation end times yielded more complex results. Prior to the start of the fall wave (August 24, 2009 or earlier), the PC strategy resulted in lower attack rates and mortality in the protected age groups (5-17 and 18-54) but higher attack rates and mortality in the other age groups (0-4 and ≥55) than either the AC or the UC strategies. The PC+ strategy yielded lower still attack rates but also the lowest overall mortality reduction. For campaigns initiated during the fall wave but prior to the epidemic peak (September 28 and October 26, respectively), the PC and PC+ strategies were superior in both attack rate and mortality reductions. During this same time frame the AC strategy was more successful at reducing attack rates than the UC strategy, with a minor exception in the 18-54 age group for which the UC strategy was favorable. This general trend likely resulted from higher AC coverage in the age groups with both the highest age-specific attack rates (5-17 year olds) and the most vulnerable age groups (0-4 year olds and ≥65). This explanation is supported by the overall success, in both attack rate reduction and mortality reduction, of the PC+ strategy: as with the PC strategy, the age group with highest age-specific attack rates is completely protected, but there is also limited protection for the most vulnerable age groups. There were no substantive differences in the outcomes of all vaccination strategies (AC, UC, PC, or PC+) when the campaigns were initiated well after the epidemic peak (on November 23).

**Table 3:** Overall and age-specific final attack rates for pH1N1 for different vaccination scenarios for *R*_0 _1.4 and an 8-week vaccine campaign length.

Vaccination start date	Vaccination strategy	Attack rate,% (% reduction vs. None)				
		
		All ages	0-4	5-17	18-54	≥55
None	None	48.3	35.1	78	47.5	24.1

24-Aug	AC	8.0 (83)	4.6 (87)	12.0 (85)	8.9 (81)	3.2 (87)
	
	UC	10.1 (79)	6.7 (81)	20.0 (74)	8.8 (81)	4.0 (83)
	
	PC	6.1 (87)	7.8 (78)	1.1 (99)	8.5 (82)	5.0 (79)
	
	PC+	4.3 (91)	5.0 (86)	0.9 (99)	6.2 (87)	3.2 (87)

28-Sep	AC	25.5 (47)	16.0 (54)	42.3 (46)	25.7 (46)	10.9 (55)
	
	UC	26.8 (44)	18.3 (48)	49.2 (37)	24.7 (48)	11.7 (51)
	
	PC	14.1 (71)	14.0 (60)	9.4 (88)	18.3 (62)	9.5 (61)
	
	PC+	12.1 (75)	12.3 (65)	7.7 (90)	15.9 (67)	8.2 (66)

26-Oct	AC	42.0 (13)	28.9 (18)	69.8 (10)	41.1 (13)	19.7 (18)
	
	UC	42.0 (13)	29.8 (15)	71.5 (8)	40.3 (15)	19.9 (17)
	
	PC	37.7 (22)	29.3 (16)	54.1 (31)	39.3 (17)	20.1 (17)
	
	PC+	36.4 (25)	28.5 (19)	52.0 (33)	38.0 (20)	19.5 (19)

23-Nov	AC	47.4 (2)	34.1 (3)	77.1 (1)	46.5 (2)	23.4 (3)
	
	UC	47.4 (2)	34.2 (2)	77.2 (1)	46.4 (2)	23.4 (3)
	
	PC	47.0 (3)	34.4 (2)	75.2 (4)	46.5 (2)	23.6 (2)
	
	PC+	46.8 (3)	34.3 (2)	74.9 (4)	46.3 (3)	23.5 (2)

Attack rate is defined here as the total number of infections. 'AC' indicates the actual vaccination coverage for pH1N1 in the GVRD, 'UC' the uniform coverage vaccination strategy, 'PC' the parents and children vaccination strategy, and 'PC+' the PC/actual sequence strategy

**Table 4:** Overall and age-specific pH1N1-attributable mortality for different vaccination scenarios for *R*_0 _1.4 and an 8-week vaccine campaign length.

Vaccination start date	Vaccination strategy	Mortality per 100,000 population (% reduction vs. None)				
		
		All ages	0-4	5-17	18-54	≥55
None	None	29.6	8.6	7.4	10.9	101.1

24-Aug	AC	4.1 (86)	1.1 (87)	1.1 (85)	2.1 (81)	12.7 (87)
	
	UC	5.2 (82)	1.6 (81)	1.9 (74)	2.0 (81)	17.1 (83)
	
	PC	5.8 (80)	1.9 (78)	0.1 (99)	1.9 (83)	21.7 (79)
	
	PC+	3.7 (87)	1.2 (86)	0.1 (99)	1.4 (87)	13.5 (87)

28-Sep	AC	13.7 (54)	3.9 (54)	4.0 (46)	6.0 (46)	44.4 (56)
	
	UC	14.9 (50)	4.5 (48)	4.6 (37)	5.7 (48)	49.4 (51)
	
	PC	11.3 (62)	3.4 (60)	0.9 (88)	4.2 (62)	40.5 (60)
	
	PC+	9.8 (67)	3.0 (65)	0.7 (90)	3.6 (67)	35.1 (65)

26-Oct	AC	24.4 (18)	7.1 (18)	6.6 (10)	9.5 (13)	81.7 (19)
	
	UC	24.8 (16)	7.3 (15)	6.7 (8)	9.3 (15)	83.8 (17)
	
	PC	24.5 (17)	7.2 (16)	5.1 (31)	9.0 (18)	84.6 (16)
	
	PC+	23.7 (20)	7.0 (19)	4.9 (33)	8.7 (20)	82.0 (19)

23-Nov	AC	28.7 (3)	8.4 (3)	7.3 (1)	10.7 (2)	97.9 (3)
	
	UC	28.8 (3)	8.4 (2)	7.3 (1)	10.7 (2)	98.2 (3)
	
	PC	29.0 (2)	8.5 (2)	7.1 (4)	10.7 (2)	99.2 (2)
	
	PC+	28.9 (2)	8.4 (2)	7.1 (4)	10.7 (3)	98.8 (2)

Attack rate is defined here as the total number of infections. 'AC' indicates the actual vaccination coverage for pH1N1 in the GVRD, 'UC' the uniform coverage vaccination strategy, 'UC' the uniform coverage vaccination strategy, 'PC' the parents and children vaccination strategy, and 'PC+' the PC/actual sequence strategy

These results are especially notable because of the difference in the overall coverage between the strategies: 47% for the AC, UC, and PC+ strategies vs 36% for the PC strategy. Similar results were observed for pandemic viruses exhibiting less transmissibility than pH1N1 (e.g., *R*_0 _= 1.2) (see Figures S1 and S2, Additional file 1 and Tables S1-S6, Additional file 1 for additional values of *R*_0_), assuming different reported age-specific mortality profiles (see Figure S3, Additional file 1), and considering only the PC strategy assuming lower coverage levels in the parents and children groups (see Figure S4, Additional file 1).

### Vaccine efficacy

**Figure 5:**
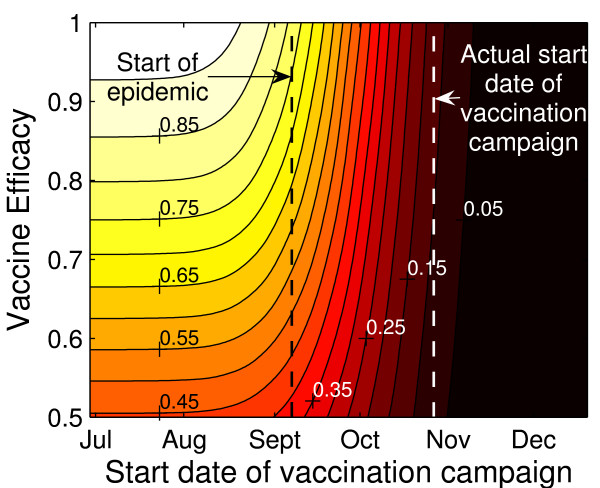
**Effect of vaccination campaign start date and vaccine efficacy on overall attack rates**. For a given vaccination campaign start date, the percent reduction in final attack rate relative to that observed in the absence of vaccination is presented for vaccine efficacy of between 50% and 100%. The ranges of the percent reduction in attack rates are indicated by solid lines and labeled. Vaccination campaigns were implemented weekly, starting July 5, 2009, with the last campaign started November 22, 2009. The start of the Vancouver influenza season on September 6, 2009 is indicated by a vertical line. All simulations assumed *R*_0 _of 1.4, latent period of 3 days, and infectious period of 7 days.

We evaluated the interplay between pH1N1 vaccine efficacy and the timing of the vaccination campaign for the Actual Coverage strategy (Figure 5, see Figure S5, Additional file 1 for sensitivity of cumulative attack rate to vaccine efficacy). If the simulated campaign begins well before the onset of the epidemic, vaccine efficacy was observed to have an important impact on depleting the size of the susceptible population and consequently reducing the outbreak size. For a vaccine with 85% or 95% efficacy, for example, the percent reduction in final attack rate relative to that observed in the absence of vaccination was 85% or 92%, respectively. However, for vaccination campaigns initiated after the onset of the epidemic, reductions in final attack rates were not highly sensitive to vaccine efficacy. For example, when vaccinations were implemented late in the epidemic stage (October or November) the percent reduction in final attack rate varied only slightly when vaccine efficacy increased from 50% to 100% (almost vertical lines representing the 5% and 15% contours in Figure 5). We observed similar patterns for other coverage scenarios (see Figure S6, Additional file 1).

### Sensitivity of results to transmission parameters for pH1N1

**Figure 6:**
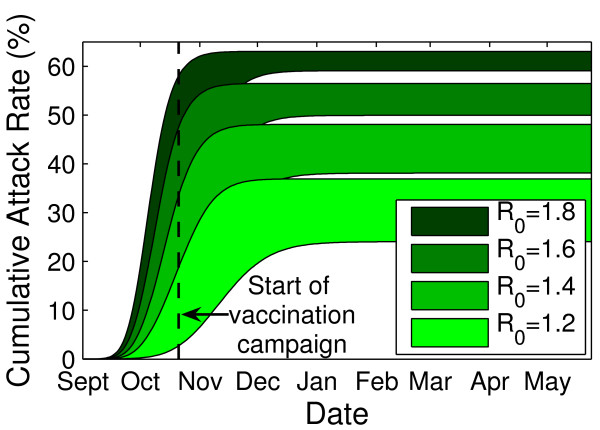
**Sensitivity of cumulative attack rates to epidemiological parameters**. Cumulative attack rates were determined for a range of values of *R*_0 _(1.2-1.8), latent period (2-4 days), and infectious period (5-7 days) in the presence of pH1N1 vaccination. The range of results observed for varying latent and infectious period lengths are presented for each value of *R*_0_. Vaccination began the week of October 26, 2009 and continued for 8 weeks, to obtain the actual coverage levels outlined in Table 2. For a given value of *R*_0_, the most steep curves (and highest attack rates in the presence of vaccination) were observed with a latent period of 2 days and infectious period of 5 days, and the least steep curves (lowest attack rates with vaccination) were observed with a latent period of 4 days and infectious period of 7 days.

Varying epidemiological parameters changed the cumulative attack rate in the presence of the Actual Coverage strategy in predictable ways. This is clear from Figure 6 (see Figure S7, Additional file 1 for results in the absence of vaccination), where we show a sensitivity analysis on the cumulative attack rate for a given *R*_0_. To generate the shaded areas, we ran simulations for parameter combinations from the ranges given in Table 1 with each combination given equal wait. We observed in particular that the lengths of shortening the infectious and latent periods resulted in more rapid epidemic growth and larger final outbreak sizes for a given value of *R*_0_, due to the shorter window of opportunity for vaccination to have an effect. Lengthening these parameters had the opposite effect. The sensitivity of final attack rates to the latent and infectious periods diminished as transmissibility (represented by *R*_0_) increased.

## Discussion

Using detailed demographic information for the GVRD, we have developed a compartmental mathematical model to estimate the transmission of pH1N1 in this population and to examine the impact of timing and age-specific coverage of different vaccination strategies for reducing the disease burden of pH1N1. Our simulations and sensitivity analyses uncovered findings with significant public health implications. First, we quantified the effect of delay in vaccine distribution relative to levels of pandemic influenza virus circulation in the population. Although vaccination is a well-established influenza preventive measure, we showed that its effectiveness during a pandemic depends greatly on the capacity to produce, distribute, and dispense vaccine in a timely manner. We demonstrated as well the importance of considering the interplay between vaccine campaign timing, demographics (especially age-specific contact rates), and the epidemiologic characteristics of the disease when developing vaccination strategies. Our sensitivity analyses verified the robustness of the results reported herein, despite the necessary inclusion of parameters in our model for which accurate estimates are currently non-existent.

We included population activity levels in our mathematical model based on a realistic representation of the contact network in the GVRD. We believe that this substantially improves the realism of the model, and gives us greater confidence in our results. For example, certain small subpopulations (e.g. health care workers or children) can have a large number of potentially disease-transmitting contacts per week, and are therefore more likely to acquire and transmit infection. Our model captures this important effect, while simplified models with homogenized activity levels would not. Age groupings addressed age-related variations in pH1N1 vulnerability to infection versus severe outcomes (mortality) each of which may constitute competing goals of the influenza immunization program. For example, vaccinating children, who tend to have higher contact rates than others, could result in a lower overall attack rate. However, as our results for the early initiation of the PC scenario showed, this strategy could leave the elderly (who experience higher mortality) relatively unprotected, thus increasing overall mortality.

In this study we included both symptomatic and asymptomatic infections in estimates of the overall attack rate. There are various estimates of the ratio of asymptomatic to symptomatic influenza cases in the literature [52,53]. More research should be directed towards conducting large-scale seroprevalence studies around the globe to reach a consensus on a plausible range corresponding to this ratio for pH1N1. When symptomatically infected, individuals may change their behaviour, deciding to stay home or cancel appointments, resulting in a reduction in their social contacts. Meanwhile, asymptomatically infected individuals may not observe such stringent self-isolation procedures but may also be less contagious owing to fewer projectile symptoms (i.e. coughing or sneezing). This effect was taken into account in the model and the related parameters were varied during sensitivity analyses. Similarly, other parameters that lack definitive parameterization in the public health literature (e.g., latent period, infectious periods) were included in the sensitivity analyses, to ensure the robustness of the reported results.

We assumed that during the herald wave in spring and early summer 2009, a relatively small fraction of the population was infected by pH1N1 symptomatically or asymptomatically. This assumption was supported by the marked difference in influenza activity in the province of BC between the two periods of April to August and September to December, based on both laboratory-confirmed cases and physicians' visit counts (see Figure S8, Additional file 1). This pattern is in contrast with the attack rate reported in other geographic areas, such as England [43,44,47], where sizable pH1N1 activity was observed in June and July. In the latter case, before comparing various immunization strategies, adjustments should be made to the assumption on the number of remaining susceptible individuals at the beginning of the second wave.

We demonstrated that while vaccine efficacy is an important factor in the outcome of vaccination before or during the early stages of an epidemic, its impact on the overall attack rate diminishes significantly when the start of the campaign approaches or passes the epidemic peak-time. Simulation results suggest that when vaccination begins near the peak of the epidemic, a 50% efficacious vaccine may reduce the overall attack rate by only 5% less than a 100% efficacious vaccine. This result, along with our findings about the importance of vaccination timing, confirm the nostrum that no matter how effective a vaccine may be in theory, it must be administered in a timely fashion to have an effect on individual or herd immune protection.

True pH1N1 infection incidence is difficult to determine, as many cases go unreported, and an unknown fraction of pH1N1 cases are asymptomatic. To support our claim that our model predictions are consistent with the epidemic, we compared the age-distribution of reported, laboratory-confirmed pH1N1 cases in the GVRD (data from the BCCDC Laboratory and BC Ministry of Health) with the age distribution of infections predicted by our model (Figure 1). We found reasonable agreement between model predictions and reported cases in age-related trends.

Our results support, to a degree, the growing modeling literature claiming that the choice of vaccination strategy can have a substantial impact on the overall attack rate of pandemic influenza. This literature largely relies on careful, detailed modeling of age structure and/or disease vulnerability levels (e.g. [32], and more recently [33,38]). The novelty we bring into this growing body of research is the incorporation of contact structure, in addition to age structure, as derived from the underlying GVRD contact network model. However, as in [37], these results also highlight the relatively greater importance of vaccination campaign timing and speed than prioritization scheme before or during the initial phase of an epidemic. Importantly, our model predicts a general equivalence of different prioritization schemes when vaccination begins at or beyond the epidemic peak.

Our results suggest that there can be two "best" targeting strategies: best given the specific vaccination campaign start time relative to the epidemic peak, and best overall given ignorance of the occurrence time of the epidemic peak (cf. Figure 4). Optimizing targeting strategies according to timing, age and disease vulnerability were carefully discussed in [18,34-36]. We leave the corresponding difficult optimization calculation using our model--which again, in contrast to previous work incorporates contact structure in addition to age structure--for future work. However we should comment that the PC strategy was chosen for comparison with the optimal strategy proposed in [18]; in there the PC strategy, when applied before the initiation of the epidemic, is the best choice in terms of both attack rate and mortality reduction. That our predictions differ may be in part due to the difference in assumptions on vaccine efficacy: while we assume equal efficacy across all age groups, Medlock et al. [18] assume that vaccines offer lesser protection in the elderly population, the population with the highest case mortality rates.

It should be noted that we assumed 100% coverage in our parents and children and parents and children/actual sequence scenarios, which may be unrealistically high. We acknowledge that this exaggerates the apparent superiority of this approach, relative to the other strategies. However this strategy has a lower overall coverage (36%) than the actual coverage or uniform coverage strategies (47%). Given the success of the PC strategy in spite of its lower overall coverage, our results therefore suggest that, for campaigns initiated before the epidemic peak, it would be worthwhile for policy-makers to consider age-based vaccine targeting strategies assuming that high coverage rates are achievable in the targeted groups. The improvements in attack rate and mortality reduction offered by the PC+ strategy, at equal coverage to AC and UC, strengthen this suggestion.

In addition, it should be noted that our results apply to the pandemic scenario where a shift in the age distribution toward greater morbidity and mortality in younger age groups is a recognized hallmark compared to seasonal influenza [14]. Our results of superior reduction in mortality with the PC strategy administered during the early rise in a pandemic wave may not apply during seasonal campaigns when attack rates are much lower and thus population mortality due to influenza is much lower for children and adults but higher for the elderly, who remain at intrinsically higher risk of severe influenza outcomes if infected.

## Conclusion

In circumstances in which vaccine production is delayed due to technological or logistical barriers, as seen with the pH1N1 vaccine, it is critical to have a good estimate of the timing of the epidemic peak before making policy decisions on vaccination strategies. Careful modeling may provide decision makers with estimates of these effects before the epidemic peak to motivate production efficiencies and inform policy decisions. Integration of real-time surveillance data with mathematical models is paramount to detect early upswings in illness activity heralding an epidemic peak and to enable public health to optimize the community benefits from proposed interventions before that occurs.

## Competing interests

The authors declare that they have no competing interests.

## Authors' contributions

*Conception and design of the study: *CJM, TA, FD, HN, PB; *Execution of mathematical simulations: *CJM, TA, MR; *Draft of the manuscript and supplementary material: *CJM, TA, FD, HN, PB; *Analysis of BC Health data: *CJM, MR, DB, EK, SD, BRC, PB; *Building Vancouver Contact Network: *DB, EK, PB; *Verification and validation of the mathematical modeling framework: *CJM, MR, PvdD, BF, MJ, ML, GA, WJ, MS, CD, PB; *Providing clinical and public health insight into analyzing the results: *FD, HN, SM, SD, BD, KJ, BR; *Providing critical input on various drafts of the manuscript: *All Authors.

## Pre-publication history

The pre-publication history for this paper can be accessed here:

http://www.biomedcentral.com/1471-2458/11/932/prepub

## Supplementary Material

Additional file 1 Click here for file
