# Supplementary material for: Vaccination against 2009 pandemic H1N1 in a population dynamical model of Vancouver, Canada: timing is everything
Source: BMC Public Health. 2011 Dec 14;11:932. doi: 10.1186/1471-2458-11-932 (PMC3280345; doi:10.1186/1471-2458-11-932)
Supplement: Additional file 1 [file 1471-2458-11-932-S1.PDF]

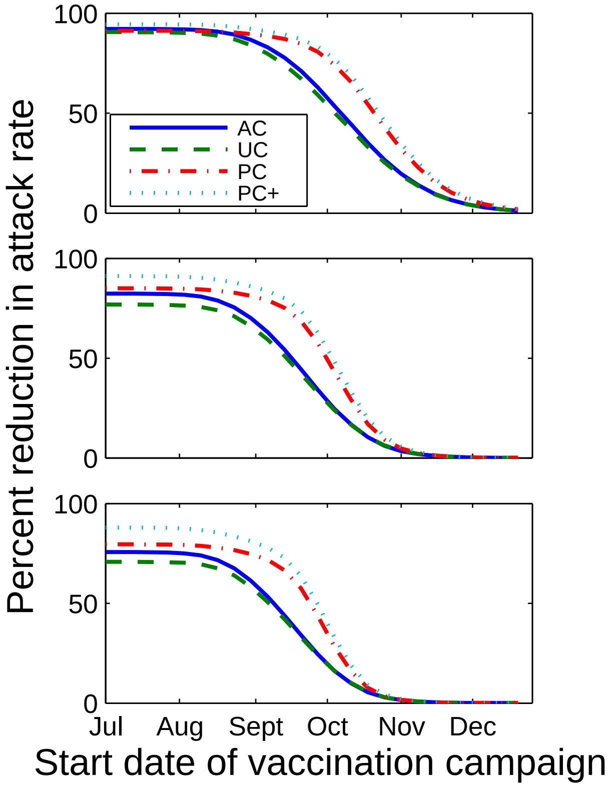

Figure S1: Impact of timing on the effectiveness of different vaccination strategies in reducing the attack rate for different values of R0. Vaccination campaigns were implemented weekly, starting July 5, 2009, with the last campaign started November 22, 2009. For a given campaign start date, the reduction in final attack rates to no vaccination was assessed using actual (blue, solid line), uniform (green, dashed line), parents and children only (red, dash-dotted line), or parents and children only/actual sequence (cyan, dotted line) vaccination strategies. R0 values are indicated in the figure. All simulations assumed latent period of 3 days, infectious period of 7 days and an 8-week vaccine roll-out period.

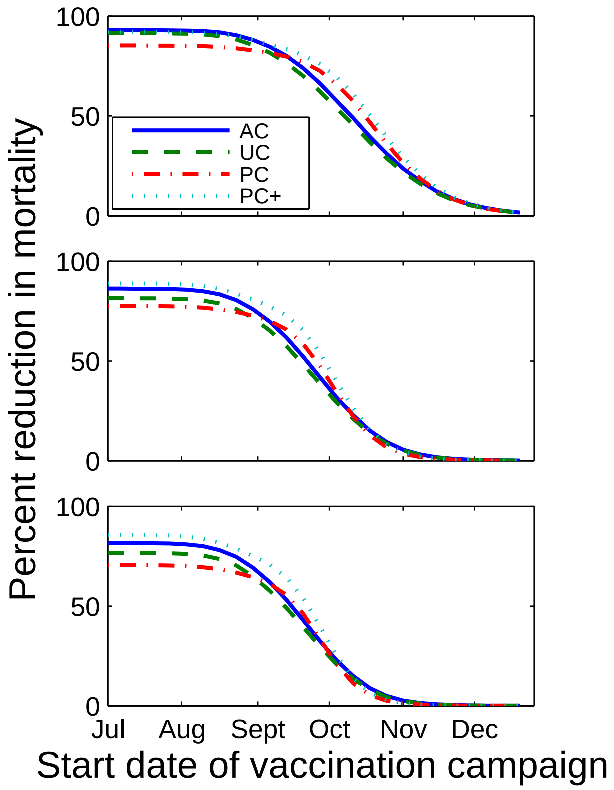

Figure S2: Impact of timing on the effectiveness of different vaccination strategies in reducing mortality for different values of R0. Vaccination campaigns were implemented weekly, starting July 5, 2009, with the last campaign started November 22, 2009. For a given campaign start date, the reduction in mortality relative to no vaccination was assessed using actual (blue, solid line), uniform (green, dashed line), or children and parents only (red, dash-dotted line), or parents and children only/actual sequence (cyan, dotted line) vaccination strategies. R0 values are indicated in the figure. All simulations assumed latent period of 3 days, infectious period of 7 days and an 8-week vaccine roll-out period.

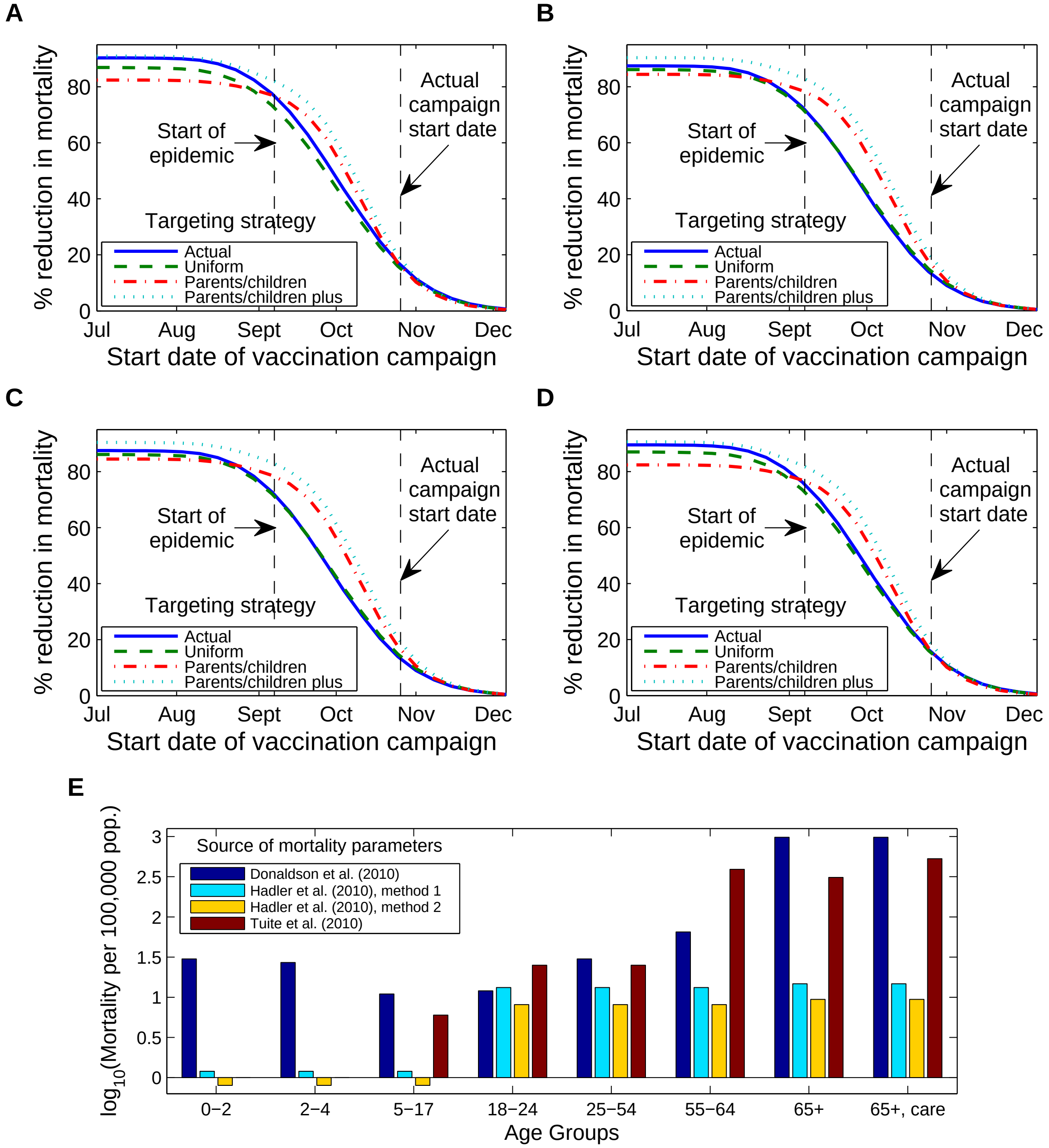

Figure S3: Impact of timing on the effectiveness of different vaccination strategies in reducing mortality for different reported age-specific mortality profiles. Vaccination campaigns were implemented weekly, starting July 5, 2009, with the last campaign started November 22, 2009. For a given campaign start date, the reduction in mortality relative to no vaccination was assessed using actual (blue, solid line), uniform (green, dashed line), or children and parents only (red, dash-dotted line), or parents and children only/actual sequence (cyan, dotted line) vaccination strategies. Mortality reduction computed from simulations using mortality rates for pH1N1 influenza reported in (a) Donaldson et al. (2010) and (b,c) Hadler et al. (2010) (39), and (d) for seasonal influenza reported in Tuite et al. (2010). Comparison between mortality profiles given in (e). All simulations assumed R0 of 1.4, latent period of 3 days, infectious period of 7 days and an eight-week vaccine roll-out period.

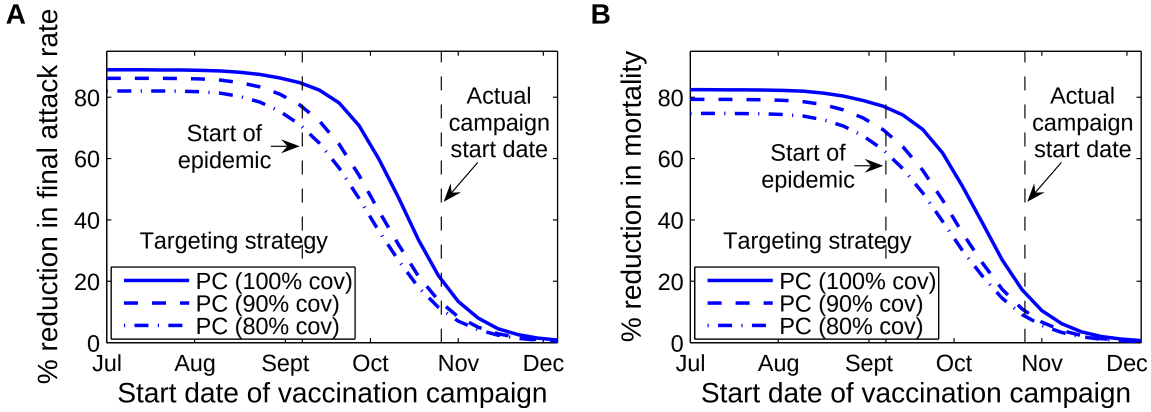

Figure S4: Impact of timing on the effectiveness of the PC strategy for different coverages levels for parents and children. Vaccination campaigns were implemented weekly, starting July 5, 2009, with the last campaign started November 22, 2009. For a given campaign start date, the reduction in (a) final attack rates and (b) mortality relative to no vaccination was assessed using the parents and children vaccination strategy at 100% (solid line), 90% (dashed line), or 80% (dash-dotted line) coverage levels. All simulations assumed R0 of 1.4, latent period of 3 days, infectious period of 7 days and an eight-week vaccine roll-out period.

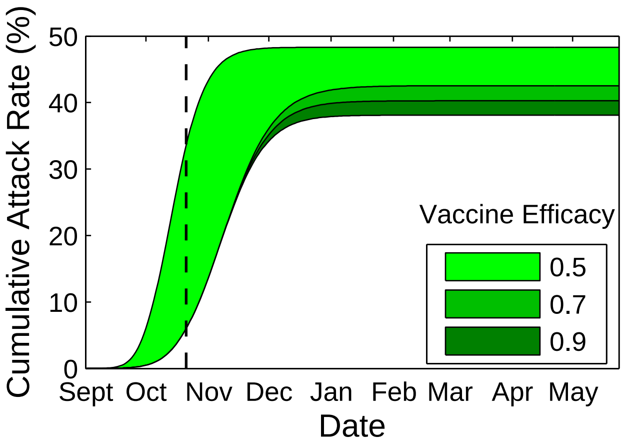

Figure S5: Sensitivity of cumulative attack rates to vaccine efficacy for baseline epidemiological parameters (see Table 1). Cumulative attack rates were determined for a range of values of R0 (1.2-1.8) in the presence of pH1N1 vaccination. The range of results observed for varying vaccine efficacy from 0.5 to 1. Vaccination began the week of October 26, 2009 and continued for 8 weeks, to obtain the actual coverage levels outlined in Table 2.

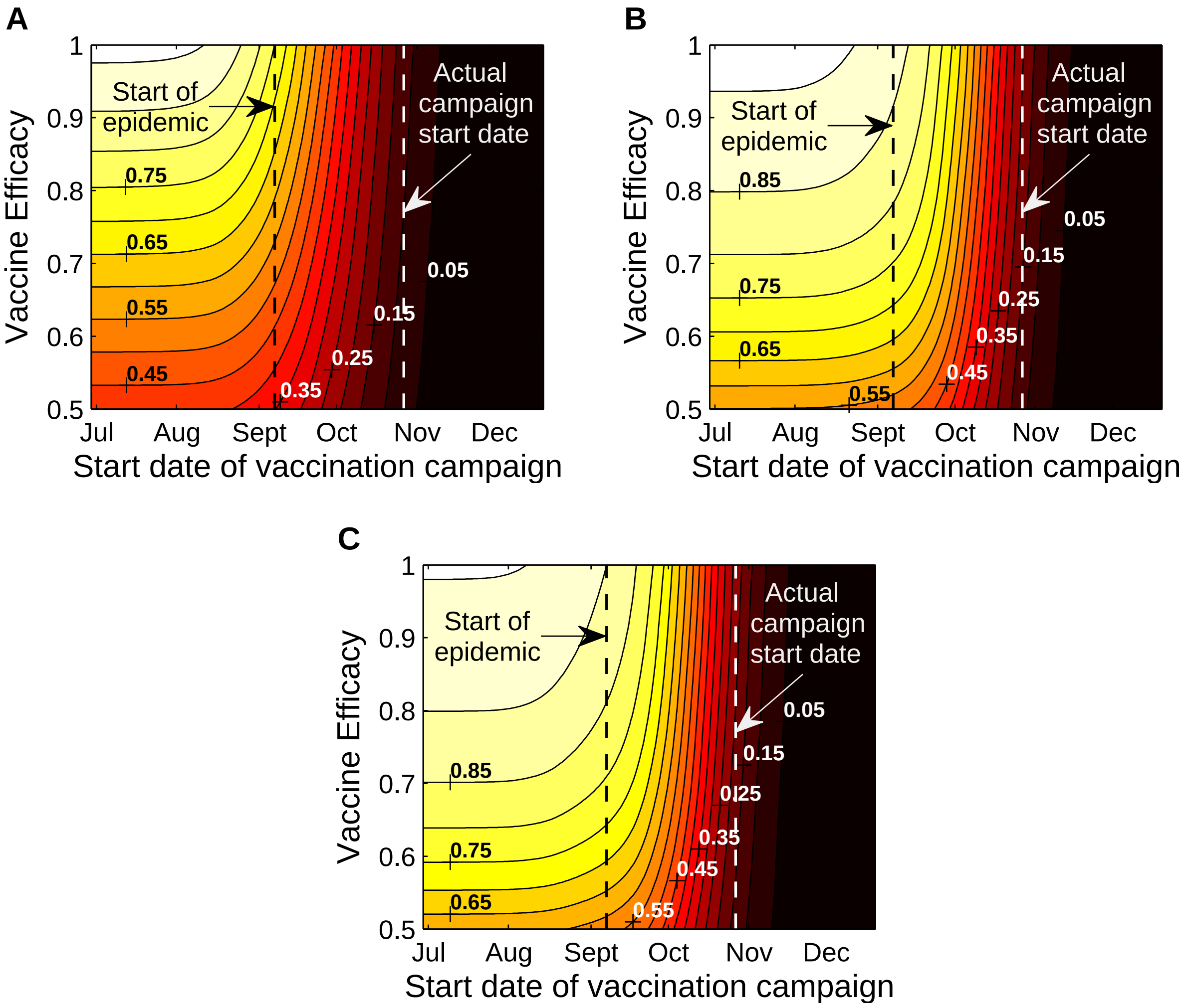

Figure S6: Effect of vaccination campaign start date on overall attack rates for (a) the uniform coverage (UC), (b) the parents-and-children (PC), and (c) the parents and children only/actual sequence vaccination strategies. For a given vaccination campaign start date, the percent reduction in final attack rate relative to that observed in the absence of vaccination is presented for vaccine efficacy of between 50% and 100%. The ranges of the percent reduction in attack rates are indicated by solid lines and labeled. 8-week vaccination campaigns were implemented weekly, starting July 5, 2009, with the last campaign started November 22, 2009. The start of the Vancouver influenza season on September 6, 2009. All simulations assumed R0 of 1.4, latent period of 3 days, and infectious period of 7 days.

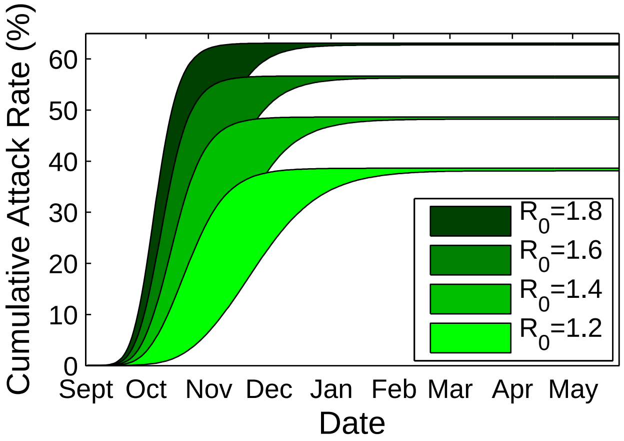

Figure S7: Sensitivity of cumulative attack rates to epidemiological parameters in the absence of vaccination. Cumulative attack rates were determined for a range of values of R0 (1.2-1.8), latent period (2-4 days), and infectious period (5-7 days) in the absence of pH1N1 vaccination. The range of results observed for varying latent and infectious period lengths are presented for each value of R0. For a given value of R0, the most steep curves (and highest attack rates in the presence of vaccination) were observed with a latent period of 2 days and infectious period of 5 days, and the least steep curves (lowest attack rates with vaccination) were observed with a latent period of 4 days and infectious period of 7 days.

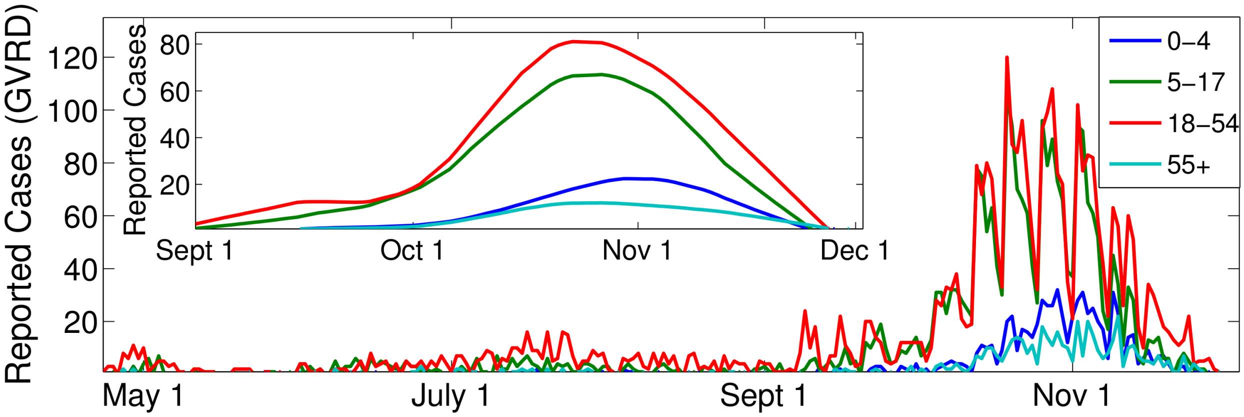

Figure S8: Confirmed daily pH1N1 cases in the GVRD reported daily, April 23-December 1st 2009. Main figure: raw data sorted according to age group. Dips are associated with delayed reporting on weekends. Inset: smoothed data (to compensate for delayed reporting) sorted according to age group, for the purposes of comparison to simulation. Data is smoothed using locally-weighted polynomial regression to smooth the confirmed cases by collection dates (Tuesdays-Fridays) and a linear interpolant to complete the curve (Saturdays-Mondays), using the software package R. Data from the BC Ministry of Health.

Table S1. Overall and age-specific pH1N1-attributable mortality for different vaccination scenarios for R_0 1.2 and an eight-week vaccine campaign length. 'AC' indicates the actual vaccination coverage for pH1N1 in the GVRD, 'UC' the uniform coverage vaccination strategy, 'UC' the uniform coverage vaccination strategy, 'PC' the parents and children vaccination strategy, and 'PC+' the PC/actual sequence vaccination strategy.

Vaccination start date	Vaccination scenario	Attack rate, % (% reduction vs. None)				
		All ages	0-4	5-17	18-54	≥55
None	None	38.2	26.4	67	36.1	17.5
24-Aug	Actual	4.1 (89)	2.5 (91)	5.9 (91)	4.5 (87)	1.6 (91)
	UC	4.9 (87)	3.4 (87)	9.8 (85)	4.3 (88)	1.9 (89)
	PC	3.7 (90)	5.0 (81)	0.7 (99)	5.1 (86)	3.0 (83)
	PC+	2.6 (93)	3.1 (98)	0.5 (99)	3.7 (90)	1.9 (89)
28-Sep	Actual	14.2 (63)	8.8 (67)	24.3 (64)	14.2 (61)	5.8 (67)
	UC	15.6 (59)	10.4 (61)	30.4 (55)	13.8 (62)	6.4 (64)
	PC	7.4 (81)	7.8 (70)	4.3 (94)	9.8 (73)	5.0 (71)
	PC+	6.4 (83)	7.0 (74)	3.5 (95)	8.5 (76)	4.4 (75)
26-Oct	Actual	28.0 (27)	18.1 (31)	50.2 (25)	26.6 (26)	11.9 (32)
	UC	28.5 (25)	19.1 (27)	53.2 (21)	26.0 (28)	12.2 (30)
	PC	21.8 (43)	17.2 (35)	30.6 (54)	23.2 (36)	11.4 (35)
	PC+	20.6 (46)	16.4 (38)	28.7 (57)	21.9 (39)	10.8 (38)
23-Nov	Actual	25.7 (7)	24.1 (8)	63.4 (5)	33.7 (7)	15.9 (9)
	UC	35.8 (6)	24.4 (7)	64.0 (4)	33.4 (8)	16.0 (8)
	PC	34.3 (10)	24.3 (8)	57.9 (13)	33.1 (8)	16.1 (8)
	PC+	33.8 (11)	24.1 (9)	57.2 (15)	32.6 (10)	15.9 (9)

Table S2. Overall and age-specific final attack rates, defined as total number of infections, for pH1N1 for different vaccination scenarios for R_0 1.6 and an eight-week vaccine campaign length. 'AC' indicates the actual vaccination coverage for pH1N1 in the GVRD, 'UC' the uniform coverage vaccination strategy, 'PC' the parents and children vaccination strategy, and 'PC+' the PC/actual sequence vaccination strategy.

Vaccination start date	Vaccination scenario	Attack rate, % (% reduction vs. None)				
		**All ages**	**0-4**	**5-17**	**18-54**	**≥55**
None	None	56.3	42.7	85	57	30.2
24-Aug	Actual	13.8 (75)	7.8 (82)	20.4 (76)	15.3 (73)	5.7 (81)
	UC	16.4 (71)	27.1 (74)	64.4 (63)	36.3 (74)	18.0 (77)
	PC	9.7 (83)	12.0 (72)	1.8 (98)	13.7 (76)	8.1 (73)
	PC+	6.8 (88)	7.7 (82)	1.4 (98)	9.9 (83)	5.1 (83)
28-Sep	Actual	37.1 (34)	24.5 (43)	58.5 (31)	38.1 (33)	17.2 (43)
	UC	37.9 (33)	27.1 (37)	64.4 (24)	36.3 (36)	18.0 (40)
	PC	23.9 (58)	22.9 (46)	17.6 (79)	30.4 (47)	16.2 (46)
	PC+	20.9 (63)	20.3 (53)	14.7 (83)	26.7 (53)	14.3 (53)
26-Oct	Actual	52.9 (6)	38.8 (9)	81.7 (4)	53.3 (6)	27.2 (10)
	UC	52.8 (6)	39.5 (8)	82.4 (3)	52.7 (8)	27.4 (9)
	PC	51.2 (9)	40.1 (6)	73.3 (14)	53.0 (7)	28.3 (6)
	PC+	50.3 (11)	39.6 (7)	71.9 (15)	52.0 (9)	27.9 (8)
23-Nov	Actual	56.0 (.6)	42.4 (.9)	84.8 (.3)	56.6 (.6)	29.9 (1)
	UC	56.0 (.6)	42.4 (.7)	84.8 (.2)	56.6 (.7)	29.9 (.9)
	PC	56.0 (.7)	42.6 (.4)	84.2 (.9)	56.7 (.6)	30.0 (.4)
	PC+	55.9 (1)	42.5 (1)	84.1 (1)	56.6 (1)	30.0 ()1

Table S3. Overall and age-specific final attack rates, defined as total number of infections, for pH1N1 for different vaccination scenarios for R_0 1.8 and an eight-week vaccine campaign length. 'AC' indicates the actual vaccination coverage for pH1N1 in the GVRD, 'UC' the uniform coverage vaccination strategy, 'PC' the parents and children vaccination strategy, and 'PC+' the PC/actual sequence vaccination strategy.

Vaccination start date	Vaccination scenario	Attack rate, % (% reduction vs. None)				
		All ages	0-4	5-17	18-54	≥55
None	None	62.8	49.4	89.6	64.7	35.7
24-Aug	Actual	20.4 (68)	11.7 (76)	29.2 (67)	22.8 (65)	8.8 (75)
	UC	22.6 (64)	15.6 (69)	40.9 (54)	21.0 (68)	10.0 (72)
	PC	14.7 (77)	17.5 (65)	2.8 (97)	20.7 (68)	12.4 (65)
	PC+	10.3 (84)	11.2 (77)	2.1 (98)	15.0 (77)	7.8 (78)
28-Sep	Actual	47.4 (24)	33.1 (33)	70.9 (21)	49.4 (24)	23.8 (33)
	UC	47.7 (24)	35.7 (28)	75.4 (16)	47.3 (27)	24.5 (31)
	PC	35.4 (44)	33.1 (33)	29.0 (68)	43.6 (33)	24.2 (32)
	PC+	31.7 (49)	30.2 (39)	24.7 (72)	39.5 (39)	21.9 (39)
26-Oct	Actual	61.0 (3)	47.1 (5)	88.3 (1)	62.7 (3)	33.9 (5)
	UC	60.9 (3)	47.6 (4)	88.6 (1)	62.3 (4)	34.0 (5)
	PC	60.6 (4)	48.4 (2)	84.7 (5)	62.9 (3)	34.9 (2)
	PC+	60.1 (4)	48.1 (3)	83.9 (6)	62.4 (4)	34.7 (3)
23-Nov	Actual	62.7 (.2)	49.3 (.3)	89.5 (.1)	64.6 (.2)	35.6 (.4)
	UC	62.7 (.2)	49.3 (.3)	89.5 (.1)	64.5 (.2)	35.6 (.3)
	PC	62.7 (.2)	49.4 (.1)	89.3 (.2)	64.6 (.2)	35.7 (.1)
	PC+	62.7 (.2)	49.4 (.1)	89.3 (.3)	64.5 (.2)	35.6 (.1)

Table S4. Overall and age-specific pH1N1-attributable mortality for different vaccination scenarios for R_0 1.2 and an eight-week vaccine campaign length. 'AC' indicates the actual vaccination coverage for pH1N1 in the GVRD, 'UC' the uniform coverage vaccination strategy, 'UC' the uniform coverage vaccination strategy, 'PC' the parents and children vaccination strategy, and 'PC+' the PC/actual sequence vaccination strategy.

Vaccination start date	Vaccination scenario	Mortality per 100,000 population (% reduction vs. None)				
		**All ages**	**0-4**	**5-17**	**18-54**	**≥55**
None	None	21.9	6.5	6.3	8.3	73.4
24-Aug	Actual	2.1 (90)	0.6 (91)	0.6 (91)	1.1 (87)	6.5 (91)
	UC	2.6 (88)	0.8 (87)	0.9 (85)	1.0 (88)	8.3 (89)
	PC	3.5 (84)	1.2 (81)	0.1 (99)	1.1 (86)	13.2 (82)
	PC+	2.2 (90)	0.8 (88)	0.1 (99)	0.8 (90)	8.0 (89)
28-Sep	Actual	7.4 (66)	2.2 (67)	2.3 (64)	3.3 (61)	23.5 (68)
	UC	8.2 (62)	2.6 (61)	2.9 (55)	3.2 (62)	26.9 (63)
	PC	6.0 (73)	1.9 (70)	0.4 (94)	2.2 (73)	21.6 (71)
	PC+	5.3 (76)	1.7 (74)	0.3 (95)	1.9 (77)	18.9 (74)
26-Oct	Actual	15.1 (31)	4.4 (31)	4.7 (25)	6.1 (26)	49.3 (33)
	UC	15.6 (29)	4.7 (27)	5.0 (21)	6.0 (28)	51.5 (30)
	PC	14.0 (36)	4.2 (35)	2.9 (54)	5.3 (36)	48.0 (35)
	PC+	13.2 (40)	4.0 (38)	2.7 (57)	5.0 (40)	45.6 (38)
23-Nov	Actual	20.0 (9)	5.9 (8)	6.0 (5)	7.8 (7)	66.6 (9)
	UC	20.2 (8)	6.0 (7)	6.0 (4)	7.7 (8)	67.3 (8)
	PC	20.1 (8)	6.0 (8)	5.5 (13)	7.6 (9)	67.7 (8)
	PC+	19.8 (9)	5.9 (9)	5.4 (15)	7.5 (10)	66.9 (9)

Table S5. Overall and age-specific pH1N1-attributable mortality for different vaccination scenarios for R_0 1.6 and an eight-week vaccine campaign length. 'AC' indicates the actual vaccination coverage for pH1N1 in the GVRD, 'UC' the uniform coverage vaccination strategy, 'UC' the uniform coverage vaccination strategy, 'PC' the parents and children vaccination strategy, and 'PC+' the PC/actual sequence vaccination strategy.

Vaccination start date	Vaccination scenario	Mortality per 100,000 population (% reduction vs. None)				
		**All ages**	**0-4**	**5-17**	**18-54**	**≥55**
None	None	36.5	10.5	8	13.1	126.8
24-Aug	Actual	7.2 (80)	1.9 (82)	1.9 (76)	3.6 (73)	22.3 (82)
	UC	8.8 (76)	2.7 (74)	2.9 (63)	3.4 (74)	28.9 (77)
	PC	9.3 (75)	2.9 (72)	0.2 (98)	3.1 (77)	34.8 (73)
	PC+	5.9 (84)	1.9 (82)	0.1 (98)	2.2 (83)	21.5 (83)
28-Sep	Actual	21.2 (42)	6.0 (43)	5.5 (31)	8.8 (33)	70.1 (45)
	UC	22.4 (39)	6.7 (37)	6.1 (24)	8.4 (36)	75.8 (40)
	PC	19.1 (48)	5.6 (47)	1.7 (79)	6.9 (47)	69.0 (46)
	PC+	16.8 (54)	5.0 (53)	1.4 (83)	6.1 (54)	60.5 (52)
26-Oct	Actual	33.1 (9)	9.5 (9)	7.7 (4)	12.3 (6)	113.6 (10)
	UC	33.4 (9)	9.7 (8)	7.8 (3)	12.1 (8)	115.2 (9)
	PC	34.0 (7)	9.9 (6)	6.9 (14)	12.2 (7)	118.9 (6)
	PC+	33.5 (8)	9.7 (7)	6.8 (15)	12.0 (9)	117.3 (8)
23-Nov	Actual	36.2 (1)	10.4 (.9)	8.0 (.3)	13.1 (.6)	125.4 (1)
	UC	36.2 (.9)	10.4 (.7)	8.0 (.2)	13.0 (.7)	125.6 (.9)
	PC	36.3 (.5)	10.5 (.4)	7.9 (.9)	13.1 (.6)	126.2 (.4)
	PC+	36.3 (1)	10.5 (1)	7.9 (1)	13.0 (1)	126.1 (1)

Table S6. Overall and age-specific pH1N1-attributable mortality for different vaccination scenarios for R_0 1.8 and an eight-week vaccine campaign length. 'AC' indicates the actual vaccination coverage for pH1N1 in the GVRD, 'UC' the uniform coverage vaccination strategy, 'UC' the uniform coverage vaccination strategy, 'PC' the parents and children vaccination strategy, and 'PC+' the PC/actual sequence vaccination strategy.

Vaccination start date	Vaccination scenario	Mortality per 100,000 population (% reduction vs. None)				
		All ages	0-4	5-17	18-54	≥55
None	None	42.7	12.2	8.4	14.9	150.3
24-Aug	Actual	10.8 (75)	2.9 (76)	2.8 (67)	5.3 (65)	34.2 (77)
	UC	12.7 (70)	3.8 (69)	3.9 (54)	4.8 (68)	42.3 (72)
	PC	14.2 (67)	4.3 (65)	0.3 (97)	4.6 (69)	53.2 (65)
	PC+	9.1 (79)	2.8 (77)	0.2 (98)	3.4 (77)	32.9 (78)
28-Sep	Actual	28.8 (33)	8.1 (33)	6.7 (21)	11.4 (23)	97.2 (35)
	UC	30.0 (30)	8.8 (28)	7.1 (16)	10.9 (27)	103.2 (31)
	PC	28.3 (34)	8.1 (33)	2.7 (68)	9.9 (33)	102.6 (32)
	PC+	25.6 (40)	7.4 (39)	2.3 (72)	9.0 (40)	92.9 (38)
26-Oct	Actual	40.6 (5)	11.6 (5)	8.3 (1)	14.5 (3)	141.9 (6)
	UC	40.8 (4)	11.7 (4)	8.4 (1)	14.4 (4)	143.1 (5)
	PC	41.6 (2)	11.9 (2)	8.0 (5)	14.5 (3)	147.0 (2)
	PC+	41.4 (3)	11.8 (3)	7.9 (6)	14.3 (4)	146.1 (3)
23-Nov	Actual	42.6 (.3)	12.1 (.3)	8.4 (.1)	14.9 (.2)	149.7 (.4)
	UC	42.6 (.3)	12.1 (.2)	8.4 (.1)	14.9 (.2)	149.8 (.3)
	PC	42.6 (.1)	12.2 (.1)	8.4 (.2)	14.9 (.2)	150.1 (.1)
	PC+	42.6 (.2)	12.1 (.1)	8.4 (.3)	14.9 (.2)	150.1 (.1)

Vaccination against 2009 pandemic H1N1 in a population dynamical model of Vancouver, Canada: timing is everything

APPENDIX

Conway JM, Tuite AR, Fisman DN, Hupert N, Meza R, Davoudi B,
English K, van den Driessche P, Brauer F, Ma J, Ancel Meyers L, Smieja M,
Greer A, Skowronski DM, Buckeridge DL, Kwong J, Wu J, Moghadas SM,
Coombs D, Brunham RC, Pourbohloul B

Contents

Appendix A Mathematical Model Details 2
- A.1 Overview 2
- A.2 Initial Condition 2
- A.3 Modeling details 3
- A.4 Equations 4

Appendix B Vaccine Distribution Rates 6
Appendix C Behaviour Change Parameter 7

Appendix A Mathematical Model Details

A.1 Overview

Our goal is to assess the impact of vaccination against pH1N1 influenza and the timing of vaccination campaigns in limiting incidence and mortality. We therefore use an extended SIR-type model, and include heterogeneity in age and behaviour to more realistically capture demographic effects.

A schematic overview of the model is given in Appendix Figure 1 where S represents those susceptible to pH1N1, E those exposed, A those in the first (asymptomatic) day of the infectious period, I those in the rest of the infectious period (symptomatic or asymptomatic infection) when behaviour may change, and M those immune. The superscripts give the vaccination class: no superscript indicates no vaccination, ^{W} indicates vaccination against pH1N1 influenza. We split the exposed and infected classes ($E_{1,2}$ or $E^W_{1,2}$, and $I_{1,2}$ or $I^W_{1,2}$ respectively) into two compartments to change the distributions of time individuals spend as exposed or infectious/possibly symptomatic, from exponential to a more realistic gamma.

Our model is heterogeneous in that we segregate the population according to age and number of weekly contacts termed degree, in an effort to capture network-type dynamics in a more computationally tractable framework. Degree groups indicate activity levels of individuals, i.e. the average number of people one would have contact within a week. We split the population into 5 degree groups: 0 to 5 contacts per week, 6 to 15 contacts per week, 16 to 30 contacts per week, 30 to 100 contacts per week, and 100+ contacts per week. The latter group represents a small fraction of the total population. It is, however, an important group; we argue that those with a higher degree (such as health care workers) are more likely to receive and transmit infection, and homogenizing over all degrees removes this important effect. We also split the population according to age, to address different susceptibilities and mortality rates from the very young to the very old, to capture the relationships between age and contact rates (school-children, for example, would necessarily have higher contact rates), and to consider the impact of variations in vaccination coverages according to age. Making these age-related distinctions is necessary to properly assess vaccination strategies. For example, vaccinating children - with their higher contact rates - could lower the overall attack rate. However, that strategy would leave the elderly - with their higher mortality rates - unprotected, thus potentially simultaneously increasing overall mortality. We use 8 different age groups: 0 to 2, 3-4, 5-18, 19-24, 25-54, 55-64, 65+ and 65+ in long-term care (we group some of these together in the results presented in the main text). In keeping with the Greater Vancouver Regional district specificity of the present study, population fractions in each age/activity level sub-compartments were derived from Vancouver data and the Vancouver network [38].

Therefore each compartment entry in Appendix Figure 1 and in the equations below corresponds to a matrix of size $N_{age} \times N_{deg}$, where N_{age} is the number of age groups, and N_{deg} is the number of degree groups (i.e. S is an $N_{age} \times N_{deg}$ matrix, as are E_1, S^W, etc). Thus, the 14 compartments and the corresponding matrix ordinary differential equations (given below) actually represent $14 * N_{age} * N_{deg}$ equations, which here is 560 equations (N_{age} = 8 and N_{deg} = 5).

A.2 Initial Condition

We set the start time of the epidemic to September 6th, which corresponds to the start of school in Vancouver. As of September 1st there were 10 confirmed cases of pH1N1 influenza in Vancouver [42]. We assumed that the number of actual cases on September 6th was 100, making a data-informed educated guess on the number of actual cases based on the number of confirmed cases.

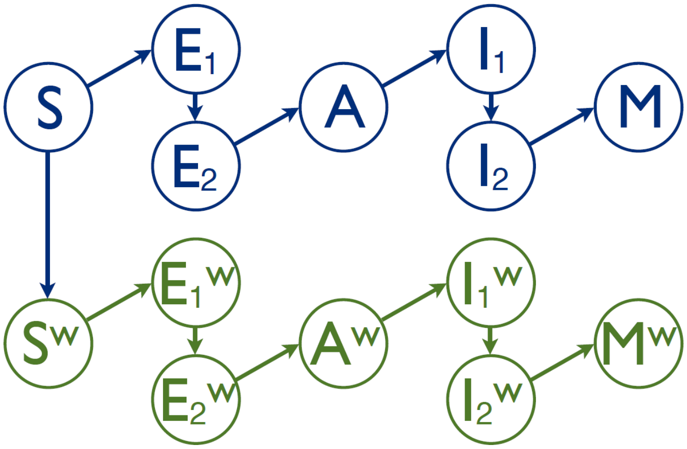

Appendix Figure 1: Schematic diagram for model with vaccination.

We then distributed them through $N_{age} \times N_{deg}$ elements of the compartment I randomly, with probability weighted according to the total population fraction in each of the $N_{age} \times N_{deg}$ elements, and the corresponding mean degree.

A.3 Modeling details

There are a few aspects of our model that require careful discussion, specifically regarding infection-transmitting contacts, vaccination, and behaviour changes.

We begin by considering infection-transmitting contacts. We use the *InfW* matrix, which gives the coupling between all the classes that can lead to infection. The $(j,k)^{th}$ entry of the *InfW* matrix is:

$$InfW_{j,k} = \sum_{rows}\sum_{cols} K.*C^{(j,k)}.*\left(A + I_1 + I_2 + P^W.*\left(A^W + I_1^W + I_2^W\right)\right)./N_{tot}$$

where $C^{(j,k)}$ is the matrix that gives contact rates of an individual of age group j , degree group k, with individuals of all the other age and degree groups, K giving the mean number of those contacts. The N_{tot} matrix gives the total population in each age and degree group. P^W indicates attenuation of infectiousness for the vaccinated: the possible decrease in transmissibility associated with pre-infection vaccination, in an individual who becomes infected in spite of being immunized. In these expressions the multiplication expression “$.*$” is used, as is the division expression “./”. These expressions do not correspond to matrix multiplication or division. Rather they correspond to term-by-term operations. For example, in the expression $F.*G = H$, the $(j,k)^{th}$ entry of H is $h_{jk} = f_{jk}g_{jk}$.

Our study centers around pH1N1 vaccination, coverages, and the timing of campaigns. We assume that there is a 2-week delay between vaccine receipt and the development of a protective immune response. This delay is implemented as a step function: 2 weeks after vaccination receipt an individual is assumed to go from unprotected to fully protected. We further assume that vaccine efficacy is imperfect - that it only offers protection 70% of the time. Vaccination rates V^W depend on the goal coverage in each age group. For the purposes of implementation, $V^W = 0$ except for during a campaign. It is derived as follows: considering only the vaccination process (i.e. ignoring the epidemic process), then for each age group j, the vaccination rate v_j^W satisfies $dS_j/dt = -v_j^W.*S_j$ such that over the campaign, the coverage cov_j is met. By solving this equation, the vaccination rate in age group j is $v_j^W = -log(1-\text{cov}_j)/(\text{length of vaccination campaign})$. This is by no means a

traditional or intuitive vaccination rate - it is designed to ensure age-specific vaccination coverages are met in a computationally efficient manner. It is, however, not entirely unrealistic: our resulting daily dose distributions (not shown in this supplement) are not logistically impossible. Further, it gives a higher daily dose distribution near the start of a campaign than near the end, which one might expect during an ordinary campaign, as perceived urgency to get vaccinated wanes. For more details see Appendix B.

We also consider behavioural change in infected individuals, through, for example, self-isolation. This is accomplished by changing the degree group of symptomatic individuals to the lowest degree group, limiting their number of contacts with other individuals. We only change the behaviour of a fraction of those in compartments I_1 or I_1^W, and I_2 or I_2^W, since (1) not all infections are truly symptomatic (some fraction of the infected remain asymptomatic throughout the infectious period), and (2) often the symptomatic only self-isolate through the worst of their symptoms, and sometimes not at all. For our purposes, at any given time, 10% of the infectious exhibit behaviour change. See Appendix C for more details on this parameter. The operators $T(\cdot)$ and $T^{-1}(\cdot)$ in the equations below indicate behavioural change in individuals while infectious and a reversion to normal behaviour when recovered.

We pose the simplification of not tracking individuals vaccinated if vaccination occurs after infection. This assumption is fair since we assumed that recovered individuals have acquired immunity, so post-infection vaccinations would not change dynamics. If tracking vaccine dose distribution were desired it could easily be implemented, but that is not a priority in this study.

A.4 Equations

Below are the ordinary differential equations at the base of the model explained above. Parameters used and their meanings are given in Appendix Tables 1 and 2. Recall that the multiplication expression ".$*$", and division expression "./", do not correspond to matrix multiplication or division. Rather they correspond to term-by-term operations. For example, in the expression $F. * G = H$, the $(j,k)^{th}$ entry of H is $h_{jk} = f_{jk} * g_{jk}$.

Parameter	Matrix	Explanation
δ_{jk}^W	D^W	Mortality rate for infected individuals
c_{jk}^{mn}	C^{mn}	Contact rate matrix - there are $N_{age} \times N_{deg}$ of these (m,n)
ρ_{jk}^W	R^W	Recovery rate
v_{jk}^W	V^W	Vaccination rate
η_{jk}^W	H^W	Rate of transition from exposed to asymptomatic
θ_{jk}^W	Θ^W	Rate of transition from asymptomatic to symptomatic

Appendix Table 1: Rate parameters. Indices indicate age group j, degree group k.

Parameter	Matrix	Explanation
γ_{jk}^{W}	G^{W}	Vaccine efficacy
β_{jk}^{W}	B^{W}	Transmissibility
ζ_{jk}^{W}	Z^{W}	Enhanced recovery for those vaccinated
κ_{jk}	K	Matrix of mean degrees
p_{jk}^{W}	P^{W}	Attenuation of infectiousness due to vaccination

Appendix Table 2: Scalar parameters. Indices indicate age group j, degree group k.

Equations for non-vaccinated individuals:

$$\begin{aligned}
\frac{dS}{dt} &= -V^{W}.*S - B^{W}.*K.*S.*InfW \\
\frac{dE_1}{dt} &= B^{W}.*K.*S.*InfW - 2H^{W}.*E_1 \\
\frac{dE_2}{dt} &= 2H^{W}.*(E_1 - E_2) \\
\frac{dA}{dt} &= 2H^{W}.*E_2 - \Theta^{W}.*A \\
\frac{dI_1}{dt} &= T\left(\Theta^{W}.*A\right) - 2R^{W}.*I_1 - D^{W}.*I_1 \\
\frac{dI_2}{dt} &= 2R^{W}.*(I_1 - I_2) - D^{W}.*I_2 \\
\frac{dM}{dt} &= T^{-1}\left(2R^{W}.*I_2\right)
\end{aligned}$$

Equations for individuals who received the vaccine:

$$\begin{aligned}
\frac{dS^{W}}{dt} &= V^{W}.*S - (1 - G^{W}).*B^{W}.*K.*S^{W}.*InfW \\
\frac{dE_1^{W}}{dt} &= (1 - G^{W}).*B^{W}.*K.*S^{W}.*InfW - 2H^{W}.*E_1^{W} \\
\frac{dE_2^{W}}{dt} &= 2H^{W}.*(E_1^{W} - E_2^{W}) \\
\frac{dA^{W}}{dt} &= 2H^{W}.*E_2^{W} - \Theta^{W}.*A^{W} \\
\frac{dI_1^{W}}{dt} &= T\left(\Theta^{W}.*A^{W}\right) - 2Z^{W}.*R^{W}.*I_1^{W} - D^{W}.*I_1^{W} \\
\frac{dI_2^{W}}{dt} &= 2Z^{W}.*R^{W}.*(I_1^{W} - I_2^{W}) - D^{W}.*I_2^{W} \\
\frac{dM^{W}}{dt} &= T^{-1}\left(2Z^{W}.*R^{W}.*I_2^{W}\right)
\end{aligned}$$

Note that the factor ‘2’ in front of the transition rate from exposed to infected H^{W} and the recovery rate R^{W} reflect the fact that there are 2 exposed classes (E_1, E_2 or E_1^{W}, E_2^{W}) and 2 infected classes (I_1, I_2 or I_1^{W}, I_2^{W}), respectively. Values for the parameters are given in the main text, except for Z^{W} which gives enhanced recovery for those vaccinated and P^{W} which gives attenuation of

infectiousness due to vaccination. These are in place for further explorations using the same model. We had no information on these parameters so for our purposes they are 1, indicating no effect. Therefore, once infected the flow through the exposed, infectious, and immune classes is the same for the vaccinated and unvaccinated.

Appendix B Vaccine Distribution Rates

If a vaccination campaign is implemented during an epidemic, as was the case for pH1N1, vaccine distribution rates may influence any results. Immunizing a higher proportion of individuals early in a campaign should result in a lower attack rate than if that same higher proportion is immunized towards the end of a campaign, for campaigns of the same length. We chose a rate of $V^W = 0$ except for during a campaign, where $v_j^W = -\log(1-\text{cov}_j)/(\text{campaign length})$, per age group j with final coverage cov_j. Scenario 1 in the bottom panel of Appendix Figure 2 is an illustration of this distribution. Our purpose is to ensure age-specific vaccination coverages are met in a computationally efficient manner (see Modeling Details), in the absence of data.

We argue that different distributions should not change the trends shown in our results. This is largely because we must consider logistics, as only so many doses can be distributed daily, which limits the height of a vaccine distribution curve. In our case, at most, a feasible 18 500 individuals (approximately .9% of the GVRD population) are vaccinated on a given day. To illustrate the minimal impact of different distribution rates we compare a few side-by-side in Appendix Figure 2. Different distribution scenarios - scenario 1 corresponding to the base case - are shown in the bottom panel. Notice that in each case the maximum number of doses distributed in a day, is of the same order of magnitude. For purposes of comparison of the role vaccination plays in the epidemic, in each case we set the campaign start at the beginning of the epidemic, when there are a maximum number of susceptibles who can be protected through immunization. Therefore the resulting incidence curves show the maximum impact different campaigns can have. These incidence curves are in the top panel of Appendix Figure 2. Notice that the overall attack rate in each case differs. In our base case (scenario 1), maximum vaccine distribution is at the start and slowly tapers through the campaign. Its resulting overall attack rate is 1.98% lower than for the scenario 2 campaign, where vaccine distribution slowly increases through a campaign; .4% lower than for the scenario 3 campaign, where most vaccinations happen in the middle; and .98% lower than the scenario 4 campaign, where the distribution rate is constant. This is not an insignificant difference (1.98% corresponds to approximately 39 600 cases in the GVRD, including unreported and asymptomatic infections); however, spread over the entire epidemic period the impact on any conclusions we would draw from results is small. Of interest also is the day-to-day difference created by varying the vaccine distribution. In the inset of the top panel we show the vaccination-associated reduction in daily incidence for each scenario. Note that for each scenario the reduction in incidence is consistently of the same order throughout the epidemic period. The maximum difference between the curves is approximately 1300 new infections in the GVRD (including unreported and asymptomatic infections), between scenarios 1 and 3. Again these differences are small enough that any conclusions we would draw from results is small.

With these illustrative examples as support, we claim that in the absence of data, our vaccine distribution term - chosen for computational ease - sufficiently captures resulting spread of infection for the purposes of understanding larger trends.

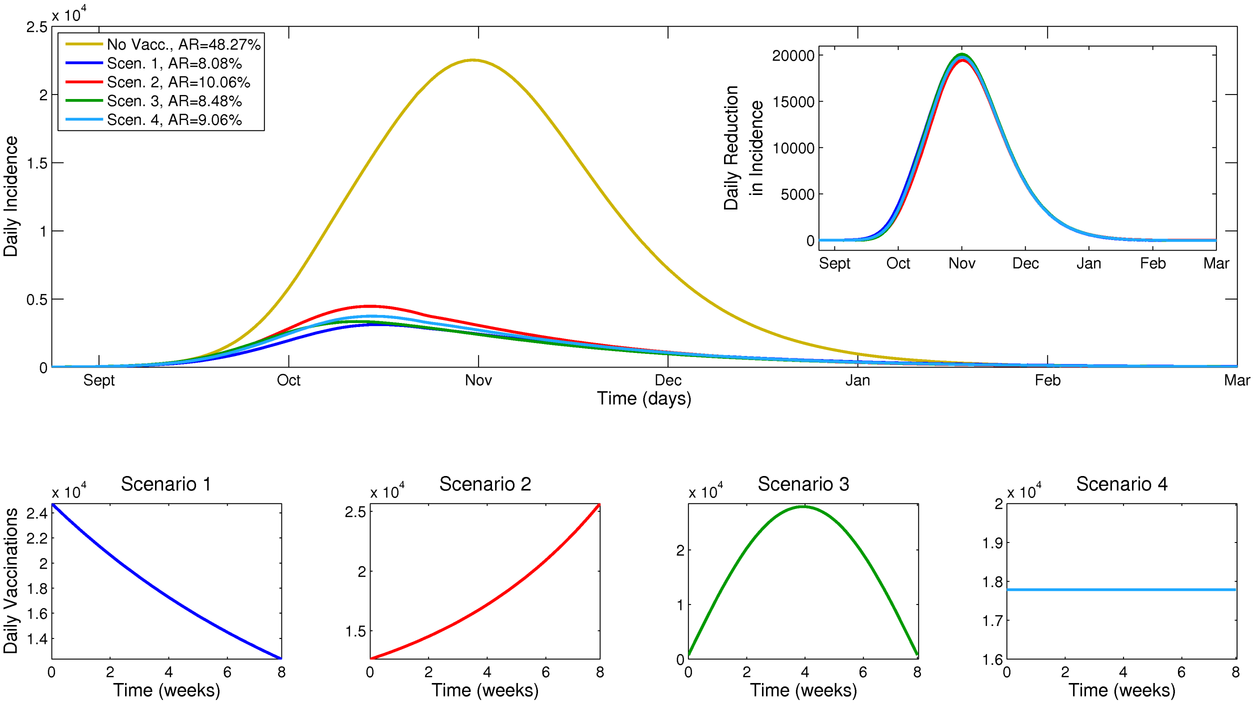

Appendix Figure 2: Vaccine distribution (bottom panels) and corresponding incidence curves (top panel), with vaccination-related reduction in incidence (inset, top panel). For these simulations we use our base case parameters, with each vaccination campaign beginning at the start of the epidemic.

Appendix C Behaviour Change Parameter

The behaviour change parameter used in the model represents the fraction of the infected (after the pre-symptomatic period) population at a given time that are self-isolating. Our baseline value for this parameter is 10%. Notice in Appendix Figure 3 that high compliance in terms of self-isolation results in dramatic changes in attack rate. Appendix Figure 3 gives results up to a parameter of 60%; it is improbable that such a high percentage reflects reality. A high fraction of infections are likely asymptomatic; studies on different influenza infections indicate that this fraction may be as high as 60% [54]. Generally, in the absence of a fever people do not self-isolate; early results from an observational study in Nova Scotia suggest that up to 41% of confirmed symptomatic infections do not include fever as a symptom [55]. Further, fevers do not last through the entire infectious period (recall that the behaviour change parameter represents the fraction of the infected population self-isolating at a given time). We therefore feel that our baseline estimate of 10% for this parameter is well-justified.

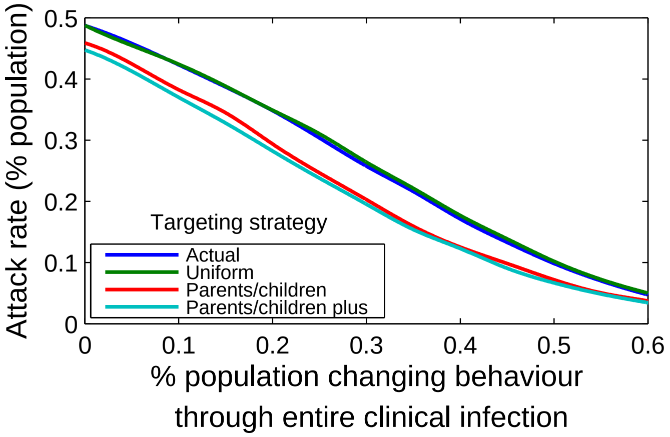

Appendix Figure 3: Sensitivity of results to behaviour change parameter. Each curve gives the final attack rate as a function of the behaviour change, using baseline parameters (see Table 1) for each of the 3 vaccination strategies.

Though attack rates vary dramatically with the behaviour change parameter, for our purposes it is important to note that the trends and modeling results reported in this paper remain qualitatively similar with variations in this parameter up to 70% (results not shown; no sensitivity analysis for the parameter higher than 70% were performed). For example, in Appendix Figure 3 though the overall attack rates for each vaccination strategy are changing, the Parent-Child plus (PC+) strategy always results in the lowest attack rate, followed closely by the Parent-Child strategy (PC), then followed by the Uniform Coverage (UC) strategy and the Actual Coverage (Actual) which are very near each other.
